# Factors Predicting Pathological Response to Neoadjuvant Chemoradiotherapy in Rectal Cancer: The Experience of a Single Institution with 269 Patients (STONE-01)

**DOI:** 10.3390/cancers13236074

**Published:** 2021-12-02

**Authors:** Michele Fiore, Pasquale Trecca, Luca E. Trodella, Roberto Coppola, Marco Caricato, Damiano Caputo, Alessandro Coppola, Gian M. Petrianni, Gabriele D’Ercole, Edy Ippolito, Rolando M. D’Angelillo, Sara Ramella

**Affiliations:** 1 Department of Radiation Oncology, Campus Bio-Medico University, 00128 Rome, Italy; p.trecca@unicampus.it (P.T.); luca.trodella@unicampus.it (L.E.T.); g.petrianni@unicampus.it (G.M.P.); g.dercole@unicampus.it (G.D.); e.ippolito@unicampus.it (E.I.); s.ramella@unicampus.it (S.R.); 2 Department of General Surgery, Campus Bio-Medico University, 00128 Rome, Italy; r.coppola@unicampus.it (R.C.); d.caputo@unicampus.it (D.C.); a.coppola@unicampus.it (A.C.); 3 Department of Colorectal Surgery, Campus Bio-Medico University, 00128 Rome, Italy; m.caricato@unicampus.it; 4 Department of Radiation Oncology, University “Tor Vergata”, 00133 Rome, Italy; d.angelillo@med.uniroma2.it

**Keywords:** rectal cancer, chemoradiation, predictive factors, response

## Abstract

**Simple Summary:**

Neoadjuvant chemoradiotherapy (CRT) followed by total mesorectal excision is currently the standard of care for locally advanced rectal cancer (LARC)**.** This retrospective cohort study evaluated the pathological response after CRT in relation to treatment factors and patient and disease factors in order to find useful indicators to further improve the efficacy of CRT and create tailored therapeutic approaches. To date, the optimal timing for surgery after CRT has not been established. In literature, there are controversial results regarding the risk of higher surgical morbidity and perioperative complications due to delayed surgery. In our study carried out on 269 consecutive LARC patients, among the items analyzed, an interval time from CRT to surgery of >8 weeks was the only independent significant factor for pCR and downstaging.

**Abstract:**

Aims: The aim of this study was to define a potential benefit of pathological complete response rate (pCR) and downstaging rate after neoadjuvant chemoradiotherapy (CRT) in relation to treatment and patient factors in locally advanced rectal cancer. Methods: We performed a retrospective cohort study. Patients were divided according to chemotherapy regimens concurrent to radiotherapy (1-drug vs. 2-drug) and according to the time interval between the end of CRT and surgery (≤8 weeks vs. >8 weeks), as well as in relation to specific relevant clinical factors. Logistic regression was used to estimate the independent factors for pCR and downstaging. Results: 269 patients were eligible for this study. Overall, pCR and downstaging rates were 26% and 75.4%, respectively. Univariate analysis showed that female gender (*p* = 0.01) and time to surgery >8 weeks (*p* = 0.04) were associated with pCR; age > 70 years (*p* = 0.05) and time to surgery >8 weeks (*p* = 0.002) were correlated to downstaging. At multivariate analysis, interval time to surgery of >8 weeks was the only independent factor for both pCR and downstaging (*p* = 0.02; OR: 0.5, CI: 0.27–0.93 and *p* = 0.003; OR: 0.42, CI: 0.24–0.75, respectively). Conclusions: This study indicates that, in our population, an interval time to surgery of >8 weeks is an independent significant factor for pCR and downstaging. Further prospective studies are needed to define the best interval time.

## 1. Introduction

Colorectal cancer is currently the fourth most common malignancy and the second cause of cancer mortality worldwide [1]. For rectal cancer, 742,139 new diagnoses and 326,982 deaths occurred in 2020. New cases will exceed one million by 2040 [2]. In the last 10 years, the mortality trend has seen a decline in the more developed countries due to early detection programs and improvement in cancer treatment management.

Neoadjuvant chemoradiotherapy (CRT) followed by total mesorectal excision (TME) is the current standard of care for locally advanced rectal cancers (LARC) and for distal tumors close to the sphincter structure in order to achieve organ preservation [3,4].

After preoperative CRT, 15 to 20 percent of pathological complete response (pCR) rates have been reported [5]. Many series show that complete and partial tumor regressions after preoperative CRT are associated with improved long-term outcomes in patients with rectal carcinoma independent of their initial clinical T and N stages [6,7,8]. A pooled analysis of 3105 patients demonstrated a 5-year DFS of 83.3 percent for patients with pCR and of 65.6 percent for those without pCR (HR 0.44, 95% CI 0.34–0.57; *p* < 0.0001) with a median follow-up of 48 months [9]. These findings suggest a more favorable prognosis for patients with pCR than those with residual disease after CRT, indicating pCR as a goal in the neoadjuvant treatment of rectal cancer [5,10].

Moreover, the possibility of a complete response to the neoadjuvant therapy has led to investigation of the hypothesis of the non-operative management (NOM) approach for patients with clinical complete response (cCR) after CRT. Prospective series and a large systematic review revealed that a “wait-and-see” policy and organ-sparing options such as local excision of the tumor may be possible alternatives for patients who achieve cCR after neoadjuvant CRT [11,12].

In the last few years, various strategies have been tested to improve the efficacy of the neoadjuvant CRT and to personalize therapies, such as chemotherapy intensification and prolonged time interval to surgery.

Chemotherapy intensification was investigated during CRT, before and after surgery. Several studies have evaluated the addition of platinum-based chemotherapy to fluoropyrimidine in combination with radiotherapy. In the CAO/ARO/AIO-04 trial, an increased compliance and higher rates of pCR rates were reached by adding oxaliplatin to CRT [13]. Instead, in PETACC-6, STAR-01 and ACCORD 12 trials, the addition of oxaliplatin did not confer additional benefit, but resulted in substantially more toxicity when added to neoadjuvant radiation therapy [14,15,16]. Therefore, the efficacy of a double drug chemotherapy during neoadjuvant radiotherapy remains unclear, and this treatment is not currently recommended [17].

More recently, neoadjuvant therapy with mFOLFIRINOX before CRT was investigated in the PRODIGE 23 phase III trial. Patients were randomly assigned to receive preoperative CRT plus surgery, followed by adjuvant chemotherapy for 6 months or 6 cycles of mFOLFIRINOX, followed by the same preoperative CRT scheme plus surgery and adjuvant chemotherapy for 3 months. The ypT0N0 rate was 11.7 percent vs. 27.5 percent respectively (*p* < 0.001) and 3-year DFS was significantly increased in the second group: 68.5 percent vs. 75.7 percent (95% CI 0.49–0.97, *p* = 0.034). A relevant increase in G4 neutropenia was recorded during CRT in the experimental arm (2.8% vs. 0% *p* = 0.02) [18].

Another open issue in rectal cancer management is the optimal timing between the end of CRT and surgery. Many retrospective studies have suggested that the tumor response is time-dependent, proposing that lengthening the interval between the end of CRT and surgery may increase the rate of pCR [19,20,21]. In contrast, the randomized controlled trial GRECCAR 6 found no difference in pCR, but rather a worse quality of mesorectal resection in the arm with an interval time from the end of neoadjuvant therapy to surgery of more than 7 weeks [22].

The aim of this study is to define the potential benefit of the pathological response after neoadjuvant CRT in LARC patients in relation to treatment factors, such as chemotherapy intensification and time interval to surgery, and to patient and disease factors, in order to find useful indicators to further improve efficacy of CRT and create tailored therapeutic approaches.

## 2. Materials and Methods

We performed a retrospective cohort study on LARC patients treated at Campus Bio-Medico University of Rome. Patient data were collected from clinical records. Patients signed informed consent to the use of their clinical data for scientific purposes. Inclusion criteria was: age > 18 years, clinical stage II (T3–4, N0) or III (any T, N1–2) invasive rectal adenocarcinoma with distal tumor border within 12 cm from the anal verge by proctoscopy. In all patients, staging was performed with a multilayer computed tomography scan (CT) of the chest, abdomen and pelvis with intravenous contrast, and pelvic magnetic resonance imaging (MRI) with intravenous contrast.

**Figure 1:**
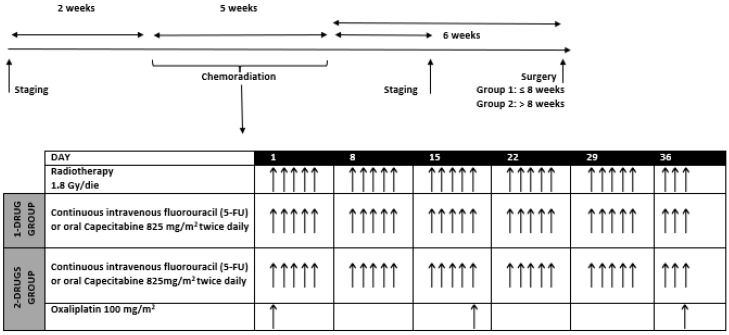
Time scheduling and concomitant chemoradiation regimen: continuous intravenous fluorouracil or oral capecitabine was given each day of radiation, for five days a week; oxaliplatin was given every 19 days.

Patients were treated using 3D conformal radiotherapy or intensity-modulated radiotherapy (IMRT). The total radiation dose was 50.4 Gy with conventional fractionation of 1.8 Gy daily, for five days a week, concurrent to chemotherapy. Figure 1 explains the time scheduling and the concomitant chemoradiation regimen.

The occurrence and nature of any adverse events were recorded in accordance with the National Cancer Institute Common Toxicity Criteria scale (version 4.0, National Cancer Institute, Bethesda, MD, USA). When multiple treatment-related adverse events of the same type occurred in the same patient, only the most severe ones were reported.

After the completion of CRT, restaging was performed. Tumor response was defined in accordance with the World Health Organization (WHO) by MRI and CT scan. Surgery was performed according to the principles of TME. Evaluation of the pathological specimens included ypTpN staging, resection margin involvement (positive if ≤1 mm distance from tumor to margin), and tumor regression grade (TRG) according to the Mandard tumor regression grade scoring system [23].

After resection, patients were evaluated according to the standard surveillance protocol of follow-up.

The primary endpoint of the study was to evaluate the pCR rate (defined as the absence of tumor cells in the surgical specimen, both at the primary tumor site and at regional lymph nodes) and the downstaging rate (defined as lower pathologic stage compared with the pre-treatment clinical stage).

Secondary endpoints were the overall survival (OS), the disease-free survival (DFS) and local control (LC).

Patients were divided into two groups according to chemotherapy regimens concurrent to radiation therapy (“1-drug” group: continuous intravenous fluorouracil (5-FU) or oral capecitabine (CAPE); “2-drug” group: 5-FU or CAPE with intravenous platinum-based chemotherapy). Patients were also stratified into two different time intervals according to the time between the end of neoadjuvant CRT and surgery (group 1: ≤8 weeks; group 2: >8 weeks). Patients were also divided according to relationship to relevant pretreatment factors as gender, age (≤70 years vs. >70 years), pre-treatment clinical stage (II vs. III) and distance of tumor from anal verge (≤5 cm vs. >5 cm).

All study sample characteristics were summarized with descriptive statistics.

Evaluation of differences was performed with log-rank test. Heterogeneity among study groups were evaluated by Pearson’s *χ*^2^ test. The level of heterogeneity indicated the variability among the groups.

Given that pCR and pathologic downstaging are known to be robust factors for early efficacy and long-term survival in LARC patients, in this study, the efficacy was measured through these two endpoints in order to assess both tumor and nodal clearance. Univariate and multivariate logistic regression analyses were used to model the pCR and the pathological downstaging probability using clinically relevant variables. Variables considered in multivariate analysis were selected by significance during univariate analysis. A two-sided *p* value of 0.05 was considered significant.

Overall survival (OS) was determined from the day of the histological diagnosis. DFS was measured from the day of surgery to the observation of progression/recurrence, or to the last follow-up if no event was observed. OS and DFS curves were calculated with the Kaplan-Meier method.

The statistical analysis was performed with the Statistical Package for Social Sciences, version 25 (SPSS Inc., Chicago, IL, USA).

The study protocol was approved by the independent ethics committee of Campus Bio-Medico University of Rome (8.19 TS ComEt CBM, 21 August 2019).

## 3. Results

### 3.1. Patients and Treatment

From July 2007 to July 2018, 564 patients diagnosed with rectal cancer were treated with radiation therapy at Campus Bio-Medico University of Rome. Of these, 362 patients underwent neoadjuvant CRT. Seventy-three patients were excluded from the final analysis for lack of data related to pathological specimens and clinical follow-up. Twenty patients did not undergo surgery after CRT and were excluded from the evaluation. Consequently, 269 patients were analyzed. The demographic and clinical characteristics of these patients are listed in Table 1. Two hundred and sixty-six patients (98.9%) completed CRT. Chemotherapy was administered concurrently with radiotherapy in all patients and consisted of 5-FU or CAPE in 144 patients (53.5%) and 5-FU/CAPE plus oxaliplatin in 125 patients (46.5%). Of 125 patients receiving a 2-drug regimen, 97 patients (77.6%) were under 70 years old (mean 59 years).

### 3.2. Toxicity

During CRT, the all-grade toxicities were hematological (anemia 52.7%, leukopenia 37.9%, neutropenia 19.5%, thrombocytopenia 32.9%) and gastrointestinal (diarrhea 50%). Grade 2 and grade 3 leukopenia and neutropenia occurred in 6% and 3.7% of patients, respectively. Grade 2 and grade 3 anemia and thrombocytopenia occurred in 9.6% and 7.7% of patients, respectively. Grade 2 diarrhea occurred in 25.2% of patients and grade 3 in 4.4%. No grade 4 toxicity was reported (Table 2).

Three patients did not complete neoadjuvant CRT, one patient due to perianal abscess and two due to genitourinary disease not related to treatment (infective cystitis and urinary retention).

In 32 patients with grade 2 and 3 gastrointestinal toxicity, the treatment was interrupted (median time of interruptions was 4 days, range 1–14 days).

As reported in Materials and Methods, patients were stratified into two different groups according to concurrent chemotherapy schedule (1-drug vs. 2-drug). Grade 2 and grade 3 acute hematological toxicity occurred in 19 patients (13.1%) who received 1-drug chemotherapy during radiotherapy and in 54 patients (43.2%) who received 2-drug chemotherapy. Grade 2 and grade 3 gastrointestinal toxicity rate was 20.1% in the first group and 40.8% in the second one (29 and 51 patients respectively).

In the 1-drug group, radiotherapy interruptions related to adverse events occurred in 18 patients (12.5%) with a median time of interruptions of 4.6 days (range 1–14 days). Of 125 patients treated with 2-drug CRT, radiotherapy interruptions occurred in 14 patients (11.2%) with a median time of interruptions of 3.2 days (range 1–5 days). Table 3 compares all-grade toxicity in the 1-drug and 2-drug groups.

### 3.3. Surgery

**Figure 2:**
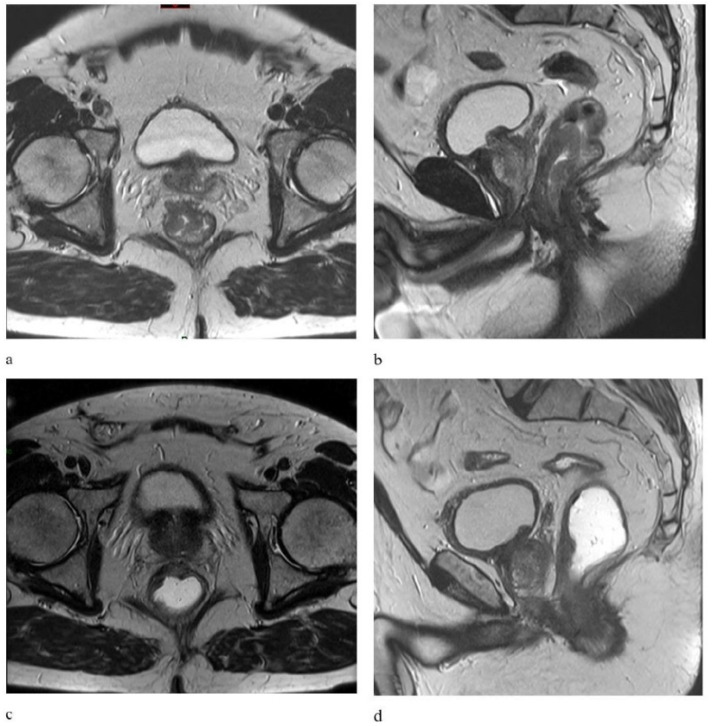
Axial and sagittal T2-weighted MR images show a distal rectal tumor with mesorectal fascia (MRF) infiltration in a patient with a clinical staged T3-N1 tumor before (**a**,**b**) and after chemoradiotherapy (**c**,**d**) showing a locally advanced rectal cancer with a good response to neoadjuvant chemoradiotherapy. MR, magnetic resonance.

Figure 2 illustrates an example of clinical response comparing pre-treatment MRI with post-CRT images.

A total of 236 patients (87.7%) were operated by low anterior resection and 33 (12.3%) by abdominal perineal resection. All patients underwent TME. R0 resection was obtained for 261 patients (97%). One hundred and seventeen patients (43.5%) had acute postoperative complications (within 30 days after surgery). For the entire population, median time from the end of CRT to surgery was 9.3 weeks (range, 1.3–46.8 weeks).

As reported in Materials and Methods, patients were stratified according to interval time to surgery.

In total, 100 patients (37.2%) underwent surgery with an interval time of ≤8 weeks and 169 patients (62.8%) with an interval time of >8 weeks. The acute postoperative complications rate was 46% (46 patients) for patients with interval time to surgery of ≤8 weeks and 42% (71 patients) for patients with interval time to surgery of >8 weeks. The most frequent causes of postoperative complications were fever (39 patients, 14.5%) and anastomotic leakages (36 patients, 13%).

Among all patients of the study, 70 (26%) underwent postoperative chemotherapy.

### 3.4. Pathological Complete Response (pCR) and Downstaging Rate

Overall, pCR rate was 26% (*n* = 70 patients). For patients who did not achieve pCR, TRG were as follows: TRG2 17.1%, TRG3 33.4%, TRG4 21.6%, TRG5 1.9%.

Heterogeneity among the study groups according to gender, age (≤70 years vs. >70 years), pre-treatment clinical stage (II vs. III), distance of tumor from anal verge (≤5 cm vs. >5), time to surgery (≤8 weeks vs. >8 weeks) and concurrent chemotherapy schedule (1-drug vs. 2-drug) was detected by Pearson’s *χ*^2^ test (Table 4).

There were no significant differences between the pCR and no-pCR groups in terms of clinical stage, distance of tumor from anal verge and concurrent chemotherapy schedule. Specifically, the pCR rate was 26.5% (38 patients) in the 1-drug group (5-FU or CAPE) and 25.6% (32 patients) in the 2-drug group (5-FU or CAPE with intravenous platinum-based chemotherapy). Of patients with TRG2, 24 were in the 1-drug group, 22 in the 2-drug group. A significant difference was found for gender (*p* = 0.012) and time to surgery (*p* = 0.029).

The pCR rate was 19% (19 patients) for patients who underwent surgery with an interval time of ≤8 weeks group and 30.1% (51 patients) for patients who underwent surgery with an interval time of >8 weeks, as shown in Table 4. At univariate analysis, gender (95% CI 1.14–3.49, *p* = 0.015) and time to surgery (95% CI 0.29–0.98, *p* = 0.04) were significantly correlated to pCR. Logistic regression showed that other variables such as age and concomitant chemotherapy schedule were not significantly associated with pCR.

The positive impacts of female gender (95% CI 1.2–3.74, *p* = 0.01) and interval time to surgery of >8 weeks (95% CI 0.27–0.93, *p* = 0.02) were confirmed at multivariate analysis, and were the only remaining correlated factors with pCR (Table 5).

A downstaging from clinical to pathological stage was obtained in 203 patients (75.4%). Table 6 and Table 7 detail pre-treatment MRI T and N stage compared with pathologic T and N stage.

The downstaging rate according to chemotherapy schedule was 76.3% and 74.4% in 1-drug group and 2-drugs group, respectively.

The downstaging rate in relation to time intervals to surgery resulted in 65% in the group with time ≤8 weeks and 81.6% in the group with time >8 weeks.

At univariate analysis for downstaging rate, only age <70 years (95% CI 0.98–3.11, *p* = 0.05) and interval time to surgery of >8 weeks (95% CI 0.23–0.73, *p* = 0.002) were significantly correlated with downstaging and, of these factors, a resting time of >8 weeks was confirmed at multivariate analysis (95% CI 0.24–0.75, *p* = 0.003) (Table 8).

The median follow-up of the entire population was 51.1 months (range, 4.1–133.9). At time of analysis, only 16 patients (6%) had a follow-up period of less than 1 year, while 119 patients (44%) had a follow-up period of longer than 5 years. A total 56 (20.8%) had a progression of disease. Patterns of failure included the following: 9 (3.4%) local recurrence, 47 (17.4%) distant metastases, 6 (2.2%) both local failure and distant metastases. The most common sites of distant metastases were lung (*n* = 22; 46.8%) and liver (*n* = 19; 40.4%). A total of 42 patients (15.6%) died; 13 deaths were tumor related, and 29 were due to intercurrent diseases. Of the remaining 227 patients, 204 (75.8%) are alive without evidence of disease.

All patients were included in survival analysis. The relationship between pCR and OS, DFS, metastasis-free survival (MFS) and local control (LC) was analyzed.

The 5-year and 10-year OS were significantly higher for patients who obtained a pCR compared with patients who do not achieve it (94% vs. 79% and 77% vs. 71%, respectively; *p* = 0.05).

pCR was also significantly correlated with 5-year and 10-year DFS, MFS and LC.

The 5-year and 10-year DFS were both 94% for patients who obtained pCR, and 69.4% and 68.1% for patients with partial response (*p* < 0.001).

The 5-year and 10-year MFS were both 94% for patients who obtained pCR, 74.5% and 73% for patients with partial response (*p* < 0.001).

No local recurrence was registered for patients who obtained a pCR. The 5-year and 10-year LC in patients who did not achieve pCR was 90.6% and 89% respectively (*p* = 0.02).

Survival outcomes were analyzed for each study group. According to the concurrent chemotherapy schedule, the 5-year and 10-year OS was 77% and 65% for the 1-drug group, and 86% and 77.2% for the 2-drug group (*p* = 0.07). In patients who underwent surgery with an interval time of ≤8 weeks, 5-year and 10-year OS was 80.7% and 69.8%, respectively, compared with 84.5% and 78% in patients with an interval time of >8 weeks (*p* = 0.05). The 5-year and 10-year DFS were 72% and 69% in the ≤8 weeks group and both 77% in the >8 weeks group (*p* = 0.2).

## 4. Discussion

In our study carried out on 269 consecutive LARC patients, among the items analyzed, interval time from CRT to surgery of >8 weeks resulted the only independent significant factor for pCR and downstaging.

Usually, surgical resection is performed after 6–8 weeks from the end of preoperative CRT based on the findings of the Lyons R90-01 trial [24]. In our experience, the pCR rate was 19% for patients who underwent surgery with an interval time of ≤8 weeks group and 30.1% in patients who underwent surgery with an interval time of >8 weeks, resulting in significance at univariate and multivariate analysis (*p* = 0.02). The increased interval may potentially increase the tumor downstaging as the radiation-induced necrosis appears to be a time-dependent effect.

These results agree with the findings of several other retrospective series that showed how a higher rate of pCR and better oncological outcomes with fewer postoperative complications are associated to a longer time interval between CRT and surgery [6,7,8,9,25,26,27,28]. An Italian multicenter retrospective cohort study of 2094 patients with LARC demonstrated a correlation between delayed surgery and pCR, reporting a 2.4 greater probability of achieving pCR in patients who underwent surgery after 16 weeks from the end of CRT compared to patients operated within 6 weeks [29]. More recently, a meta-analysis was performed including studies that compared standard time to surgery (<8 weeks from the end of CRT) and delayed surgery (≥8 weeks). A total of 26 publications (4 randomized trials, 1 prospective interventional study and 20 observational retrospective studies) for a total of 25,445 patients were analyzed: an interval time of <8 weeks was significantly related with pCR (*p* = 0.001) without increasing postoperative complication rates [30].

Moreover, in our study, a correlation between an increased resting time and the pathological response to CRT was also observed. The downstaging rate was 81.6% in the group with the interval time to surgery of >8 weeks and 65% in the group with time to surgery ≤8 weeks. At univariate analysis for downstaging, only age (*p* = 0.05) and interval time to surgery (*p* = 0.002) were significant, and the resting time of >8 weeks was confirmed at multivariate analysis (*p* = 0.003).

In several series that have investigated the efficacy of a prolonged time for surgery, pathological findings revealed a higher rate of tumor and nodal downstaging when analyzing them separately [26,31,32,33]. Our study, evaluating the pathological stage, is the first that obtains a significantly higher rate of overall downstaging in the group of the delayed surgery. This analysis may be even more useful if we consider the possibility to identify patients with a good prognosis to better personalize their treatment.

To date, the optimal timing for surgery after CRT has not been established. In literature, there are controversial results regarding the risk of higher surgical morbidity and perioperative complications due to delayed surgery. The GRECCAR 6 trial is the only phase III randomized study in favor of standard surgery and is considered to have developed the most robust data on the increased risk of complications with delayed surgery. Two hundred and sixty-five patients were randomized to be operated 7 or 11 weeks after the end of CRT. The authors in their conclusions reported a higher postoperative morbidity rate and a worse quality of mesorectal excision in the group who had delayed surgery without improvement of the pCR rate. However, with regard to the severe complications observed (grade III–V Clavien-Dindo scale), there was no difference between the two groups: 14.4% (18/125 patients vs. 14.8% (19/128 patients) in the 7-week and 11-week groups, respectively, and the only two patients with grade V toxicity were in the 7-week group (5%, 2/125 vs. 0%, 0/128). In addition, anastomotic leakage was more frequent in the 7-week group compared with the 11-week group: 15.9% vs. 14%. The pCR rate for the delayed surgery group was 17.4% compared with 15% of the 7-week group—a difference not statistically significant [22].

In our experience, the delayed surgical approach did not increase the risk of surgical complications. Overall, acute postoperative complications rates were similar for patients with an interval time to surgery of ≤8 weeks and patients with an interval time to surgery of >8 weeks: 46% and 42% respectively.

Similarly, Garcia-Aguilar et al. in a phase II study on 259 patients, extended considerably the resting time between CRT and surgery by administering two, four or six cycles of chemotherapy after CRT. They did not observe increased complications or technical difficulties, reporting higher pCR rates with an increased waiting time after CRT: 18% in the control group, 25% after 2 cycles of chemotherapy, 30% after 4 cycles and 38% after 6 cycles [34].

In our study, in patients who underwent surgery with an interval time of ≤8 weeks, 5-year and 10-year OS were 80.7% and 69.8%, compared with 84.5% and 78% in patients with an interval time of >8 weeks (*p* = 0.05). The 5-year and 10-year DFS were 72% and 69% in the ≤8 weeks group, and 77% in the >8 weeks group (*p* = 0.2).

Interestingly, we found that a resting time of >8 weeks may be considered a safe approach regarding the risk of tumor progression. The possibility to have both a better knowledge of the optimal time to obtain the highest rate of complete response and clearer evidence that a longer interval to surgery is not detrimental in terms of survival outcomes may be important evidence supporting the feasibility and safety and of an organ preservation strategy.

Chemotherapy intensification has also been investigated in our study as a well-known potential strategy to increase the number of patients achieving pCR.

In this study, chemotherapy was administered concurrently with radiotherapy in all patients and consisted of 5-FU or CAPE in 144 patients (53.5%), and 5-FU or CAPE plus oxaliplatin in 125 patients (46.5%). The pCR rate was similar in both groups (26.5% vs. 25.6% *p* = 0.88). Grade 2 and grade 3 acute hematological toxicity occurred in 19 patients (13.1%) who received 1-drug chemotherapy during radiotherapy and in 54 patients (43.2%) who received 2-drug chemotherapy. Grade 2 and grade 3 gastrointestinal toxicity rate was 20.1% in the first group and 40.8% in the second group. In our study, the addition of oxaliplatin during preoperative radiotherapy resulted in a higher toxicity rate in the absence of clinical benefits.

Similar results have been reported in several randomized trials. Among these, in the STAR-01 phase III trial, 747 patients with LARC were randomly assigned to receive pelvic radiation and concomitant 5-FU alone or in association with oxaliplatin. The rate of pCR was 16% in both arms (*p* = 0.904). Grade 3–4 adverse events during CRT were higher in the 2-drug group (24% vs. 8%; *p* = 0.001) [14].

Allegra et al. randomized 1608 patients affected by LARC to receive 5-FU or CAPE during CRT with or without oxaliplatin. No statistically significant differences were found in terms of DFS, LC and OS between the groups. Moreover, the addition of oxaliplatin did not reduce local recurrence even in the highest-risk patient population, whereas grade 3–4 diarrhea was statistically significantly higher in patients who received oxaliplatin (*p* < 0.0001) [13].

In the ACCORD 12 trial, 598 patients were randomly assigned to receive capecitabine or capecitabine plus oxaliplatin concurrently with preoperative radiotherapy. The rate of pCR did not differ significantly (*p* = 0.09), nor did the rate of complete and near complete response (*p* = 0.008) [15].

The addition of oxaliplatin during radiotherapy is currently being investigated in several ongoing clinical trials [35,36]. Data of our analysis support the current indication that a platinum-based chemotherapy during preoperative radiotherapy in rectal cancer is not recommended.

The main limitation of this study is the retrospective design, while the strengths of the study are the homogeneity of the population in terms of staging and treatments and the long period of follow-up.

## 5. Conclusions

In conclusion, in our cohort, an interval time from CRT to surgery of >8 weeks is the only significant factor for pCR and downstaging.

The findings of this study may be useful for data collection and for encouraging the development of future trials that could help to better clarify how radiotherapy can further improve clinical outcomes in LARC patients.

## Figures and Tables

**Table 1:** Patient and disease characteristics.

Characteristics	No of Patients (All = 269)	%
Age (years)		
Median	65.6	-
Range	34–88	-
Sex		
Male	178	66.1
Female	97	33.9
Clinical T stage		
T2	39	14.5
T3	201	74.7
T4	29	10.8
Clinical N stage		
N0	75	27.8
N1	127	47.2
N2	64	23.7
N3	3	1.3
Clinical stage		
II	75	27.8
III	194	72.2
Distance from anal verge (cm)		
<5	92	34
5–10	151	56
>10	26	10

**Table 2:** All grade toxicity during chemoradiotherapy.

Toxicity	Grade 1	Grade 2	Grade 3	Grade 4
	*n* (%)	*n* (%)	*n* (%)	*n* (%)
Hematological				
Anemia	116 (43.1)	23 (8.5)	3 (1.1)	0 (0)
Leukopenia	86 (31.9)	12 (4.5)	4 (1.5)	0 (0)
Granulocytopenia	43 (15.8)	6 (2.2)	4 (1.5)	0 (0)
Thrombocytopenia	68 (25.2)	20 (7.4)	1 (0.3)	0 (0)
Gastrointestinal				
Diarrhea	55 (20.4)	68 (25.2)	12 (4.4)	0 (0)

**Table 3:** All grade (G) toxicity in the two groups (1-drug vs. 2-drug chemotherapy).

Toxicity	1-Drug	2-Drug	1-Drug	2-Drug	1-Drug	2-Drug
	G1	G1	G2	G2	G3	G3
	*n* (%)	*n* (%)	*n* (%)	*n* (%)	*n* (%)	*n* (%)
Hematological						
Anemia	51 (35.4)	65 (52)	9 (6.2)	14 (11.2)	0 (0)	3 (2.4)
Leukopenia	30 (20.8)	56 (44.8)	4 (2.8)	8 (6.4)	0 (0)	4 (3.2)
Granulocytopenia	10 (6.9)	33 (26.4)	4 (2.8)	2 (1.6)	0 (0)	4 (3.2)
Thrombocytopenia	29 (20.1)	39 (31.2)	2 (1.3)	18 (14.4)	0 (0)	1 (0.8)
Gastrointestinal						
Diarrhea	29 (20.1)	26 (20.8)	23 (15.9)	45 (36)	6 (4.2)	6 (4.8)

**Table 4:** pCR rate according to patients’ characteristics and treatment factors.

Variables	All	pCR	no-pCR	Pearson’s *χ*^2^ Test
		*n* (%)	*n* (%)	
		70 (26%)	199 (74%)	
Male	178	38 (21.3)	140 (78.7)	*p* = 0.012
Female	91	32 (35.2)	59 (64.8)	
Age ≤ 70	181	53 (29.3)	128 (70.7)	*p* = 0.053
Age > 70	88	17 (19.3)	71 (80.7)	
Stage II	75	20 (26.6)	55 (73.4)	*p* = 0.4
Stage III	194	50 (25.7)	144 (74.3)	
Tumor Location ≤ 5 cm	130	32 (24.6)	98 (75.4)	*p* = 0.3
Tumor Location > 5 cm	139	38 (27.3)	101 (72.6)	
1-Drug	144	38 (26.4)	106 (73.6)	*p* = 0.4
2-Drug	125	32 (25.6)	93 (74.4)	
Interval Time ≤ 8 weeks	100	19 (19)	81 (81)	*p* = 0.029
Interval Time > 8 weeks	169	51 (30.1)	118 (69.9)	

pCR, pathologic complete response; no-pCR, non-pathologic complete response.

**Table 5:** Results of univariate and multivariate analysis for pCR.

Variables	Analysis for pCR					
	Univariate			Multivariate		
	OR	95% CI	*p* Value	OR	95% CI	*p* Value
Sex						
Male	1.99	1.14–3.49	0.01	2.12	1.20–3.74	0.01
Female						
Age (years)						
≤70	1.72	0.93–3.21	0.08	-		
>70						
CT schedule						
1-Drug	1.04	0.6–1.8	0.88	-		
2-Drug						
Time to surgery (weeks)						
≤8	0.54	0.29–0.98	0.04	0.5	0.27–0.93	0.02
>8						

**Table 6:** Pre-treatment T stage compared with pathologic T stage (*n* = 269).

Pre-Treatment Staging	Postoperative Staging				
	pT0	pT1	pT2	pT3	pT4
cT2 (*n* = 39)	9	6	13	11	0
cT3 (*n* = 201)	57	13	49	80	2
cT4 (*n* = 29)	8	1	6	11	3
Total (*n* = 269)	74	20	68	102	5

**Table 7:** Pre-treatment N stage compared with pathologic N stage (*n* = 269).

Pre-Treatment Staging	Postoperative Staging		
	pN0	pNx	pN+
cN0 (*n* = 75)	56	6	13
cN+ (*n* = 194)	142	4	48
Total (*n* = 269)	198	10	61

pN0: ≥12 lymph nodes retrieved at intervention with no tumor invasion; pNx: <12 lymph nodes retrieved at intervention with no tumor invasion; pN+: at least one lymph node with tumor invasion.

**Table 8:** Results of univariate and multivariate analysis for downstaging.

Variables	Analysis for pCR					
	Univariate			Multivariate		
	OR	95% CI	*p* Value	OR	95% CI	*p* Value
Sex						
Male	1.03	0.57–1.85	0.92	-		
Female						
Age (years)						
≤70	1.75	0.98–3.11	0.05	1.70	0.95–3.05	0.074
>70						
CT schedule						
1-Drug	1.11	0.63–1.94	0.70	-		
2-Drug						
Time to surgery (weeks)						
≤8	0.41	0.23–0.73	0.002	0.42	0.24–0.75	0.003
>8						

## Data Availability

Research data are stored in an institutional repository and will be shared upon request to the corresponding author.
